# Chimeric Mice with Competent Hematopoietic Immunity Reproduce Key Features of Severe Lassa Fever

**DOI:** 10.1371/journal.ppat.1005656

**Published:** 2016-05-18

**Authors:** Lisa Oestereich, Anja Lüdtke, Paula Ruibal, Elisa Pallasch, Romy Kerber, Toni Rieger, Stephanie Wurr, Sabrina Bockholt, José V. Pérez-Girón, Susanne Krasemann, Stephan Günther, César Muñoz-Fontela

**Affiliations:** 1 Bernhard Nocht Institute for Tropical Medicine, Hamburg, Germany; 2 German Center for Infection Research (DZIF), Partner Site Hamburg, Hamburg, Germany; 3 Heinrich Pette Institute, Leibniz Institute For Experimental Virology, Hamburg, Germany; 4 Institute for Neuropathology, University Medical Center Hamburg-Eppendorf, Hamburg, Germany; Icahn School of Medicine at Mount Sinai, UNITED STATES

## Abstract

Lassa fever (LASF) is a highly severe viral syndrome endemic to West African countries. Despite the annual high morbidity and mortality caused by LASF, very little is known about the pathophysiology of the disease. Basic research on LASF has been precluded due to the lack of relevant small animal models that reproduce the human disease. Immunocompetent laboratory mice are resistant to infection with Lassa virus (LASV) and, to date, only immunodeficient mice, or mice expressing human HLA, have shown some degree of susceptibility to experimental infection. Here, transplantation of wild-type bone marrow cells into irradiated type I interferon receptor knockout mice (IFNAR^-/-^) was used to generate chimeric mice that reproduced important features of severe LASF in humans. This included high lethality, liver damage, vascular leakage and systemic virus dissemination. In addition, this model indicated that T cell-mediated immunopathology was an important component of LASF pathogenesis that was directly correlated with vascular leakage. Our strategy allows easy generation of a suitable small animal model to test new vaccines and antivirals and to dissect the basic components of LASF pathophysiology.

## Author Summary

Lassa fever is an arenaviral hemorrhagic fever that causes high morbidity and mortality in West Africa. Unfortunately, the lack of immunocompetent small animal models of disease has precluded understanding of the basic pathophysiology mechanisms of Lassa fever. Here we show how transplantation of a wild-type hematopoietic system into type I interferon deficient recipient mice results in a lethal mouse model of disease. Importantly, experimental infection of these chimeric mice reproduced key features of Lassa fever in humans and underscored the important role of T cells on Lassa fever-associated immunopathology.

## Introduction

Lassa fever (LASF) is a severe zoonotic viral infection caused by Lassa virus (LASV), an Old World arenavirus whose natural reservoir is the multimammate rat *Mastomys natalensis*. In endemic West-African countries such as Sierra Leone, Nigeria, Guinea and Liberia, LASF causes yearly epidemics responsible for three hundred thousand cases and more than five thousand deaths [1,2]. Clinically, LASF ranges from mild disease with non-specific signs such as malaise, asthenia, fever and myalgia to severe hemorrhagic fever, usually with the involvement of liver and kidney failure, edema of the face and neck, hypovolemia, hypoxemia, and in some cases mucosal bleeding [3]. However, the pathophysiological mechanisms responsible for these very different disease manifestations are poorly understood.

There is indirect evidence that the host immune response plays an important role in the severity of LASF. Two proteins encoded by the virus, the nucleoprotein (NP) and the Z protein, have been shown to disrupt host innnate immunity by several mechanisms leading to inhibition of the type I interferon (IFN-I) antiviral response [4–6]. The ability of LASV to inhibit IFN-I has also been linked to the capacity of the virus, at least *in vitro*, to prevent activation of monocyte-derived dendritic cells (DCs) [6,7]. While these findings provide proof of LASV antagonism *in vitro*, there is no evidence that virus-mediated immunosupression mediates LASV immunopathology *in vivo*. On the contrary, accumulating research suggests exacerbated host immune responses as a key component of LASF [3,8,9].

In humans recovering from LASF very little humoral response is observed. The neutralizing antibody titers are low and only detectable months after recovery [10,11]. Thus, it has been long thought that recovery from LASF in humans is associated with effective T cell responses [9,12]. This hypothesis has been further strengthened by experimental LASV infections in non-human primates, which indicated that recovery from LASF was associated with early detection of circulating antigen-experienced T cells [13]. In addition, a wealth of data from inbred mice infected with the prototypic old-world arenavirus lymphocytic choriomeningitis virus (LCMV), have demonstrated an important role of CD8 T cells on early control of LCMV infection in this model system [14]. Paradoxically, poor early control of LASV replication results in subsequent T cell-mediated immunopathology, a phenomenon that, at least in LCMV models, has been related to accumulation of CD8 T cells refractory to immune regulation, which in turn, promote vascular permeability [15]. In addition, mice expressing human HLA-A2 are susceptible to LASV due to poor control of early viral replication and late T cell-mediated immunopathology [8]. These previous findings raised the question of whether this deleterious effect was restricted to a particular HLA or was a pathophysiological mechanism of LASF.

A major hurdle to provide answers to these questions and investigate this general area, is that basic reseach into LASV immunology and pathogenesis has been greatly impaired by the lack of small immunocompetent animal models of disease. This is a similar case with other hemorrhagic fevers such as those caused by filoviruses and flaviviruses. With the exception of CBA mice inoculated intracranially [16], inbred laboratory mice are highly resistant to experimental infection with LASV, and only knockout mice with immune-associated deficiencies such as IFN-I receptor knockout (IFNAR^-/-^), and Signal Transducer and Activation of Transcription (STAT)-1 knockout mice, have been shown to be susceptible to LASV [17,18]. Despite the applicability of these models as *in vivo* platforms to test antiviral treatments, immunodeficient mice cannot be used to dissect the mechanisms of LASV immunity. In this study, this research question was addressed by developing a mouse model that was susceptible to LASV with intact hematopoietic immunity. Transplantation of wt bone marrow progenitor cells into irradiated IFNAR^-/-^ mice, rendered them susceptible to LASV. The infection was highly lethal and associated with viremia, liver damage and edema. In addition, immunopathology was a key component of LASF pathogenesis, was dependent on CD8 T cells and was directly correlated with vascular leakage. Thus, bone marrow transplantation allowed the establishment of an *in vivo* model retaining competent hematopoietic-driven immunity and which reproduced key features of LASF pathophysiology.

## Materials and Methods

### Ethics statement

This study was carried out in strict accordance with the recommendations of the German Society for Laboratory Animal Science and under the supervision of a veterinarian. The protocol was approved by the Committee on the Ethics of Animal Experiments of the City of Hamburg (permit no. 125/12). All efforts were made to minimize the number of animals used for experiments and to alleviate suffering of animals during experimental procedures. All staff carrying out animal experiments passed an education and training program according to category B or C of the Federation of European Laboratory Animal Science Associations. The animal experiments in this study are reported according to the ARRIVE guidelines (Kilkenny C, Browne WJ, Cuthill IC, Emerson M, Altman DG, Improving bioscience research reporting: the ARRIVE guidelines for reporting animal research. PLoS Biol. 2010;8: e1000412) A total of 145 mice were used for this study and all mice were included in the analysis.

### Viruses

LASV strains AV, Ba289, Nig04-10, Nig-CSF and Morogoro virus (MORV) strain 3017/2004 have been isolated in our laboratory and have been passaged ≤ 3 times before their use in this study. LASV strains Ba366 and Josiah, Mopeia virus strain AN21366 (MOPV) and Mobala virus strain 3099 (MOBV) have been obtained from another lab with an unknown passage history and have been passaged in our laboratory ≤ 3 times before use in this study. All virus stocks have been grown on Vero E6 cells, quantified by immunofocus assay and stored at -80°C until use. All experimental infections described in this study were performed within the biosafety level 4 (BSL4) facility at the Bernhard Nocht Institute for Tropical Medicine in Hamburg in accordance with institutional safety guidelines. Personnel wore appropriate protective equipment (biosafety suits).

### *In vitro* infectivity assays

Murine bone marrow derived macrophages and monocytes were obtained from wild type C57BL/6 mice. Eight to twelve week-old female mice were euthanized and bone marrow from tibiae and femurs was harvested as described elsewhere. Red blood cells were lysed (BD Pharm Lysing Buffer) and cells were seeded with a density of 10^6^ cells/ml in 6-well plates. For differentiation into dendritic cells, cell culture medium (RPMI with 10% FCS) was supplemented with 50 ng/ml of GM-CSF (PreProtech) or with GM-CSF conditioned media from X63-GM-CSF cells. For differentiation of bone marrow progenitors into macrophages, the medium was supplemented with M-CSF conditioned media from L929 cells. Cells were infected with a multiplicity of infection (MOI) of 0.01 with different arenaviruses as indicated, and daily samples were taken for up to 7 days post-infection. Infectious viral particles were quantified using immunofocus assay as previously described [19].

### Mice and bone marrow chimeras

IFNAR^-/-^ mice (C57Bl/6 background) were obtained from the Friedrich Loeffler Institute, Isle of Riems, Germany, and bred in the Specific Pathogen Free animal facility of the Bernhard Nocht Institute for Tropical Medicine. C57BL/6J and CD45.1^+^ congenic B6 mice, CD11b-DTR (B6.FVB-Tg(ITGAM-DTR/EGFP)_34_Lan/J), and CD11c-DTR (B6.FVB-Tg(ITGAX-DTR/EGFP)_57_Lan/J) mice were purchased from Jackson Laboratories and bred at the Heinrich Pette Institute animal facility. Bone marrow chimeric mice were generated at the Heinrich Pette Institute animal facility. Four to eight week old female mice were irradiated twice (550 rad, 4 hr apart by a Caesium source) and reconstituted with 2x10^6^ bone marrow cells from donor mice as previously described [20]. Split irradiation reduced the number of animals succumbing to irradiation to under 3%. Three to five mice per group were kept together in individually ventilated cages (IVC) and had *ad libitum* access to food and water. Engraftment in peripheral blood was evaluated 4 weeks after reconstitution by flow cytometry and the experiments were performed 4–5 weeks after transplantation. Chimeric mice showing reconstitution of donor hematopoietic cells above 85% were selected for the experiments. More than 90% of chimeric mice generated had this level of donor engraftment or higher.

### Experimental arenavirus infections

Five to thirteen animals per group were infected intraperitoneally (i.p.) with 1000 focus forming units (FFU) of LASV or MORV in 100 *μ*l DMEM containing 2% FCS. Infected mice were monitored daily for signs of disease and body weight and rectal body temperature (Thermometer BIO-TK8851 with BIO-BRET-3 rectal probe) were measured daily. Animals with severe signs of disease such as seizures, bleeding, abdominal distention, diarrhea, agony, temperature < 28°C or weight loss > 20% were euthanized as per our approved protocol guidelines. For evaluation of clinical chemistry and viremia, 30–50 μl of blood was drawn by tail vein puncture every 3–7 days over a period of 21 days (≤ 6 blood samples). When criteria for euthanasia were fulfilled or at the end of the experiment, animals were euthanized with an isoflurane overdose followed by cervical dislocation. Organs were collected from 1–3 randomly chosen mice per experimental group of 5 or more mice at day 7 post infection (p. i.) and from all mice that were euthanized between days 7–9 p. i. Organs were evaluated for the presence of infectious virus particles.

### *In vivo* cell depletion

CD4 and/or CD8 T cells were depleted by i.p. administration of monoclonal antibodies YTS191 (anti-CD4, BioXcell) and YTS169 (anti-CD8, BioXcell) on days -3 and -1 of infection. Isotype depletion control was done using the antibody LFT-2 (BioXcell). On each day, 300 μg of the respective antibody was administered. The efficiency of the depletion was verified on the day of infection (day 0) by flow cytometry and was > 98%. Specific depletion of CD11b^+^ and CD11c^+^ cells in DTR chimeric mice was accomplished by i.p injection of 0.2 μg of diphtheria toxin (DT) on days -1, 0, 1 and 2 of infection.

### Virus titrations

Organ samples were homogenized in 1 ml of DMEM with 2% FCS using Lysing Matrix D (MP Biomedical) in a beat mill. Infectious virus particles in cell culture supernatants, blood and organ samples were determined by immune focus assay using a monoclonal anti-LASV nucleoprotein antibody (2F1) for detection of infected foci as previously described [17].

### Clinical chemistry

For quantification of serum aminotransferases, serum samples were diluted 1:10 or higher in 0.9% NaCl and determined by using commercially available assays (Reflotron, Roche diagnostics). The parameters were measured for individual mice. The limit of detection for aspartate aminotransferase (AST) at 25°C was 2,25 U/l in undiluted samples. The normal range for mice was determined in 20 uninfected mice and was 40–60 U/l.

### Flow cytometry

For flow cytometry experiments mice were euthanized on days 0, 4 or 7 p. i. and spleens and lungs were collected for analysis. Single cell suspensions were prepared by cutting tissues into small fragments followed by enzymatic digestion for 45 min at 37°C with Collagenase D (2mg/ml) (Roche) in RPMI-1640 medium. Tissue fragments were further disrupted by passage through a 70-mm nylon cell strainer (BD Biosciences). Red blood cells were lysed with BD Pharm Lysing Buffer (BD Biosciences). Fc receptors were blocked with CD16/CD32 Fc Block antibody (BD Biosciences) followed by staining with fluorochrome-conjugated antibodies. A FACS LSR II or LSR Fortessa instrument (BD Biosciences) was used for flow cytometry acquisition. Analysis of data was done with FlowJo Software (Treestar).

### Histology and immunohistochemistry

Mouse tissues were fixed in 4% formalin/PBS and were embedded in paraffin. Sections (2μm) were stained with hematoxylin-eosin (H/E) or processed for immunohistochemistry as follows: After dewaxing and inactivation of endogenous peroxidases (PBS/3% hydrogen peroxide), antibody specific antigen retrieval was performed. Sections were blocked (PBS/10% FCS) and afterwards incubated with primary antibodies for rat anti-mouse CD3 (T-cells; Serotec), rabbit anti-Iba1 (monocytes/macrophages; Wako Pure Chemical Industries), B220 (B-cells; eBioscience), iNOS (inducible nitric oxide synthase; Abcam), Ki67 (cell proliferation; Abcam), cleaved caspase 3 (apoptosis marker; R&D systems) or Ly6G (neutrophils; BD Bioscience) Bound primary antibodies were detected with anti-mouse, anti-rabbit or anti-rat Histofine Simlpe Stain MAX PO immune-enzxme polymere (Nichirei Biosciences) and stained with 3,3′-Diaminobenzidine (DAB) substrate using the ultraView Universal DAB Detection Kit Ventana). Tissues were counterstained with hematoxylin. For fluorescence double labeling, primary antibodies were visualized using species-specific Cy3- or Cy2-conjugated secondary antibodies (all from Jackson ImmunoResearch Laboratories Inc.) with DAPI (Sigma-Aldrich) as nuclear staining.

### Assessment of vascular leakage

Mice were injected intravenously with 100 μl of Evans Blue (2% in NaCl). Thirty minutes post-injection mice were euthanized and perfused transcardially with 30 ml of NaCl (0.9%) containing 2 U/l heparine. Lung and liver were collected and homogenized in formamide. Evans blue was extracted in 3 ml formamide by overnight incubation at room temperature. Samples were inactivated by adding 4% formaldehyde and Evans blue absorption was measured at 650 nm.

### Luminex multiplex assay

Analysis of cytokine concentrations was done using Milliplex MAP Mouse CD8 T Cell Magnetic Bead Panel Premix 15 Plex (MCD8MAG48K-PX15) according to the manufacturer’s instructions. A Luminex 200 system (Millipore) was used for data acquisition.

### Statistical analysis

Statistical analyses were done using Graphpad Prism 6 software. Differences in survival rate were analyzed using Fisher’s exact test. Differences in weight, temperature, AST activity or virus titer in blood on individual days were analyzed using Mann-Whitney non-parametric test. Differences in plasma cytokine levels among multiple groups were evaluated via non-parametric Kruskal-Wallis test followed by Dunn’s post-test. Differences in virus titers in organs were analyzed using multiple t tests. Differences in the cellularity of immune cells over time were analyzed using a Two-Way ANOVA followed by a Bonferroni’s post-test. Statistical significance was depicted as follows:

n.s. (not significant) p > 0.05
*         p ≤ 0.05
**          p ≤ 0.01
***        p ≤ 0.001
****         p ≤ 0.0001

## Results

### Chimeric mice are susceptible to LASV

**Fig 1:**
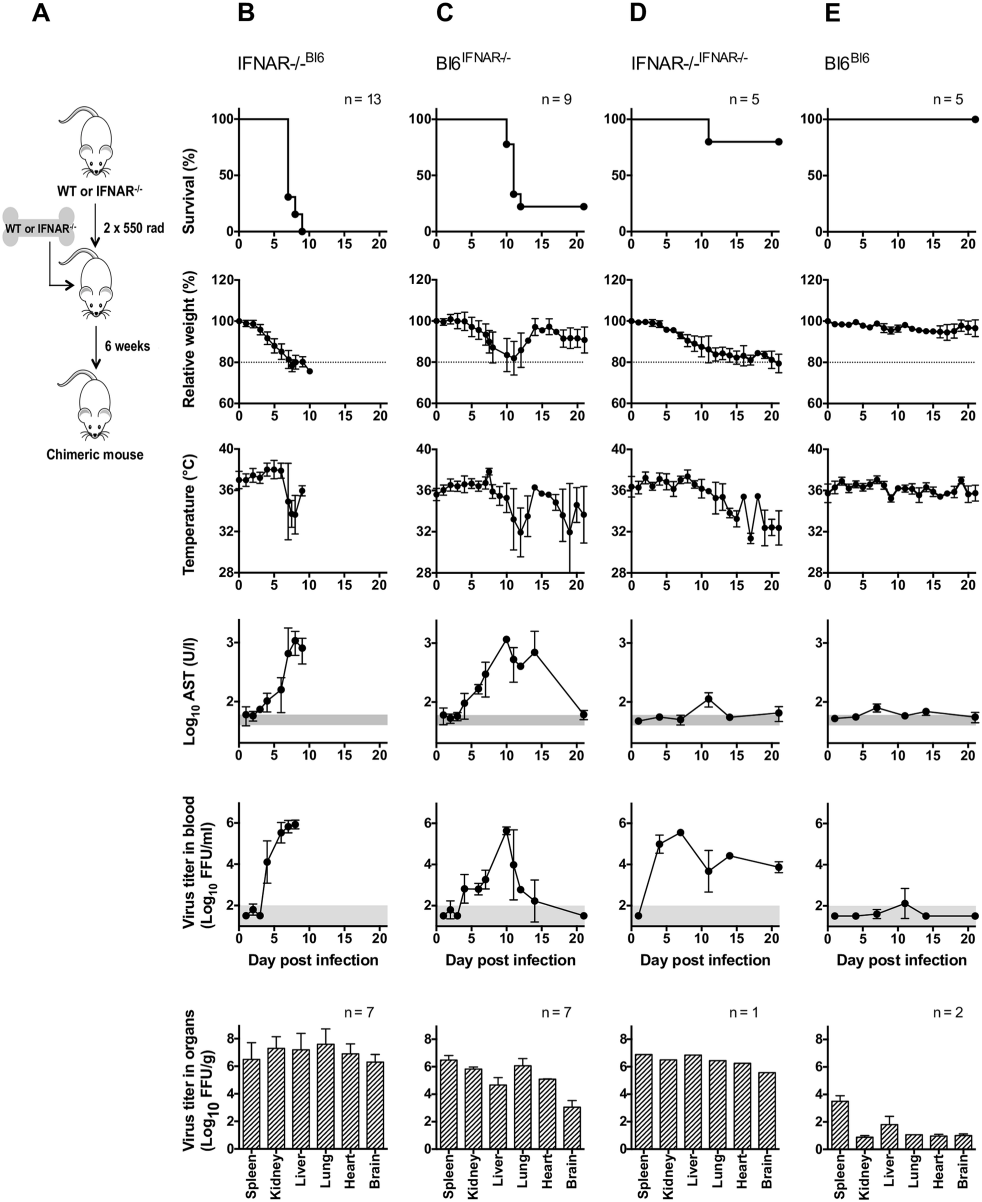
Chimeric mice are susceptible to infection with LASV. Scheme of the generation of chimeric mice (A). Chimeric IFNAR^-/- Bl6^ mice (B), Bl6 ^IFNAR-/-^ mice (C), IFNAR^-/- IFNAR-/-^ mice (D) or Bl6 ^Bl6^ mice (E) were inoculated i.p with 1,000 FFU with LASV Ba366. Survival, relative weight loss, changes in temperature, AST activity and viremia in blood and in organs were measured. For the determination of virus titers in organs, representative mice of each group were sacrificed on day 7 p.i. The normal range for AST and the limit of detection for the virus titer in blood are shaded in gray. Mean and standard deviation are shown.

While immunocompetent C57BL/6 mice are resistant to LASV, IFNAR^-/-^ mice of the same genetic background show LASV-associated morbidity, with transient weight loss and viremia [17]. To evaluate the effect of competent hematopoietic immunity on LASF pathogenesis, we generated mice in which IFN-I signaling deficiency was restricted to the radio-resistant cell compartment, namely, mostly cells of stromal origin. IFNAR^-/-^ mice were irradiated and transplanted with bone marrow progenitor cells isolated from wt C57BL/6 donor mice (Fig 1A). To ensure the greatest number of donor-derived circulating T cells, recipient mice were irradiated with split maximum radiation doses. Experimental infections were performed 4–5 weeks after transplantation in mice with more than 85% of hematopoietic donor cell engraftment (S1A Fig). Under these conditions, it is estimated that less than 1% of circulating T lymphocytes are host-derived [21]. In these chimeric IFNAR^-/- B6^ mice, LASV infection was 100% lethal, and displayed indicators of severe disease such as high serum levels of aspartate aminotransferase (AST), viremia, loss of core temperature and rapid weight loss (Fig 1B).

Next, the effect of LASV infection was tested in the reverse chimeric mouse model, in which B6 mice were transplanted with IFNAR^-/-^ bone marrow donor cells. Infection of chimeric B6 ^IFNAR-/-^ mice with LASV resulted in 75% lethality which was marked by significant weight loss in those animals who died, as well as high viremia, elevated levels of AST, viral replication in peripheral organs, and significant decrease of body temperature. Surviving mice were able to clear virus from the blood, gain weight and recover homeostatic control of the body temperature (Fig 1C).

To rule out effects associated with the generation of chimeric mice, the effect of LASV infection was assessed in wt mice transplanted with syngenic wt bone marrow (B6 ^B6^) as well as IFNAR^-/-^ mice transplanted with IFNAR^-/-^ bone marrow (IFNAR^-/- IFNAR-/-^). All B6 ^B6^ chimeric mice survived infection showing no signs of morbidity despite low levels of viral replication in peripheral organs (Fig 1E). However, the IFNAR^-/- IFNAR-/-^ chimeras showed elevated viremia and high virus replication in all organs tested (Fig 1D). Strikingly, despite ongoing systemic infection and failure of clearing virus from the blood, IFNAR^-/- IFNAR-/-^ chimeras showed only mild signs of disease with moderated weight loss and 80% survival. These results mimicked those described in knockout IFNAR^-/-^ mice, indicating that chimeric mice infected with LASV reproduced the disease observed in non-transplanted mice with the same genotype. Of note, all chimeric mice lacking IFN-I signaling in any compartment showed similar levels of viremia and virus replication in peripheral organs (Fig 1B, 1C and 1D), which indicated that virus replication alone was not responsible for LASV-associated morbidity. Non-infected IFNAR^-/- B6^ and B6 ^IFNAR-/-^ control chimeras did not show any pathological features ruling out effects of transplantation in the observed phenotype (S1B Fig). Moreover, these findings could be reproduced with representative LASV strains from lineages II, III and IV, which ruled out effects associated with a particular LASV strain (S2 Fig).

### Chimeric mice show signs of infection-associated immunopathology

**Fig 2:**
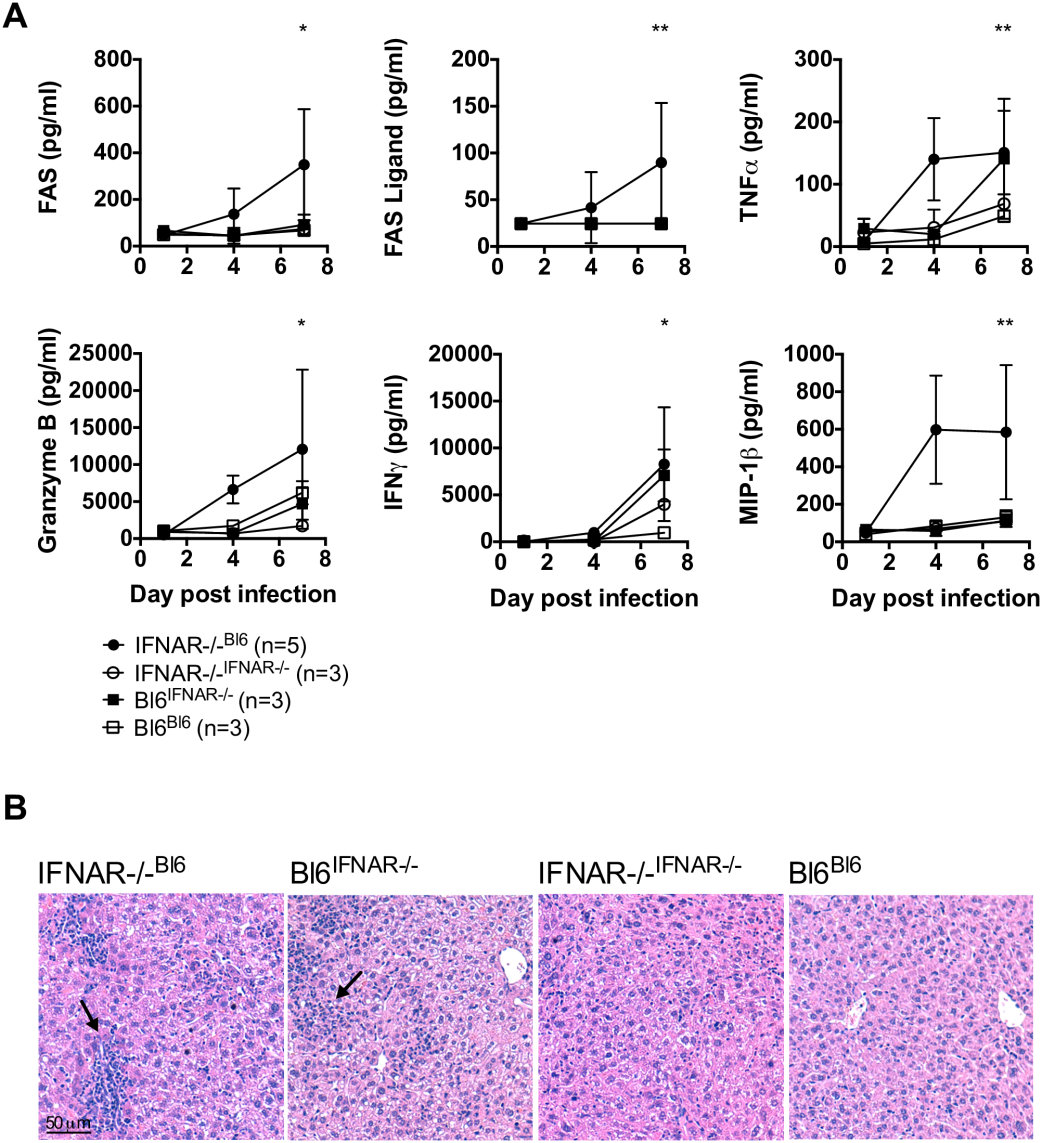
Chimeric mice show signs of infection-associated immunopathology. (A) Chimeric IFNAR^-/- Bl6^ mice, Bl6^IFNAR-/-^ mice, IFNAR^-/- IFNAR-/-^ mice or Bl6^Bl6^ mice were inoculated i.p with 1,000 FFU with LASV Ba366. Plasma concentration of FAS, FAS ligand, TNFα, Granzyme B, IFNγ and MIP-1β were analyzed at the indicated time points post infection via Luminex assay. Mean and standard deviation are shown. Differences between the indicated groups were analysed via non-parametric Kruskal-Wallis test followed by Dunn’s post-test. (B) Chimeric IFNAR^-/- Bl6^ mice, Bl6 ^IFNAR-/-^ mice, IFNAR^-/- IFNAR-/-^ mice or Bl6 ^Bl6^ mice were inoculated i.p with 1,000 FFU with LASV Ba366. Seven days post infection liver tissue sections were processed for H&A staining. The arrows indicate areas of lymphocytic infiltration.

LASV susceptibility in our model was not restricted to IFNAR^-/- B6^ chimeras since B6 ^IFNAR-/-^ mice were also highly susceptible to LASV infection with 75% lethality. We hypothesized that IFN-I competence in either the radio-resistant or the hematopoietic compartment was sufficient to provide pro-inflammatory cues that would result in disease-associated immunopathology. To test this hypothesis, the levels of serum pro-inflammatory cytokines were measured in all four chimeric mouse models at the peak of LASV infection. Significantly higher levels of TNF-α and IFN-γ were observed in both IFNAR^-/- B6^ and B6 ^IFNAR-/-^ chimeras in comparison with control chimeras (Fig 2A). Interestingly, T cell cytotoxicity markers such as FAS, FAS-L and granzyme B, as well as pro-inflammatory chemokines such as MIP-1β, were significantly upregulated in IFNAR^-/- B6^ chimeras in agreement with a more severe disease in this model and suggesting a putative role of T cells on LASV immunopathology. Further supporting immunopathology as a key component of disease, histopathological analysis of liver sections in all chimeric models showed evidence of lymphocytic infiltrates clustering in the liver of IFNAR^-/- B6^ and B6 ^IFNAR-/-^ mice, but not in control chimeras (Fig 2B). Taken together, our results indicated that chimeric mice with competent IFN-I signaling in either the radio-resistant or the hematopoietic compartment, reproduced main features of severe LASF and suggested an important involvement of host-mediated immunity in LASF pathogenesis.

### Chimeric mice are resistant to non-pathogenic arenaviruses

**Fig 3:**
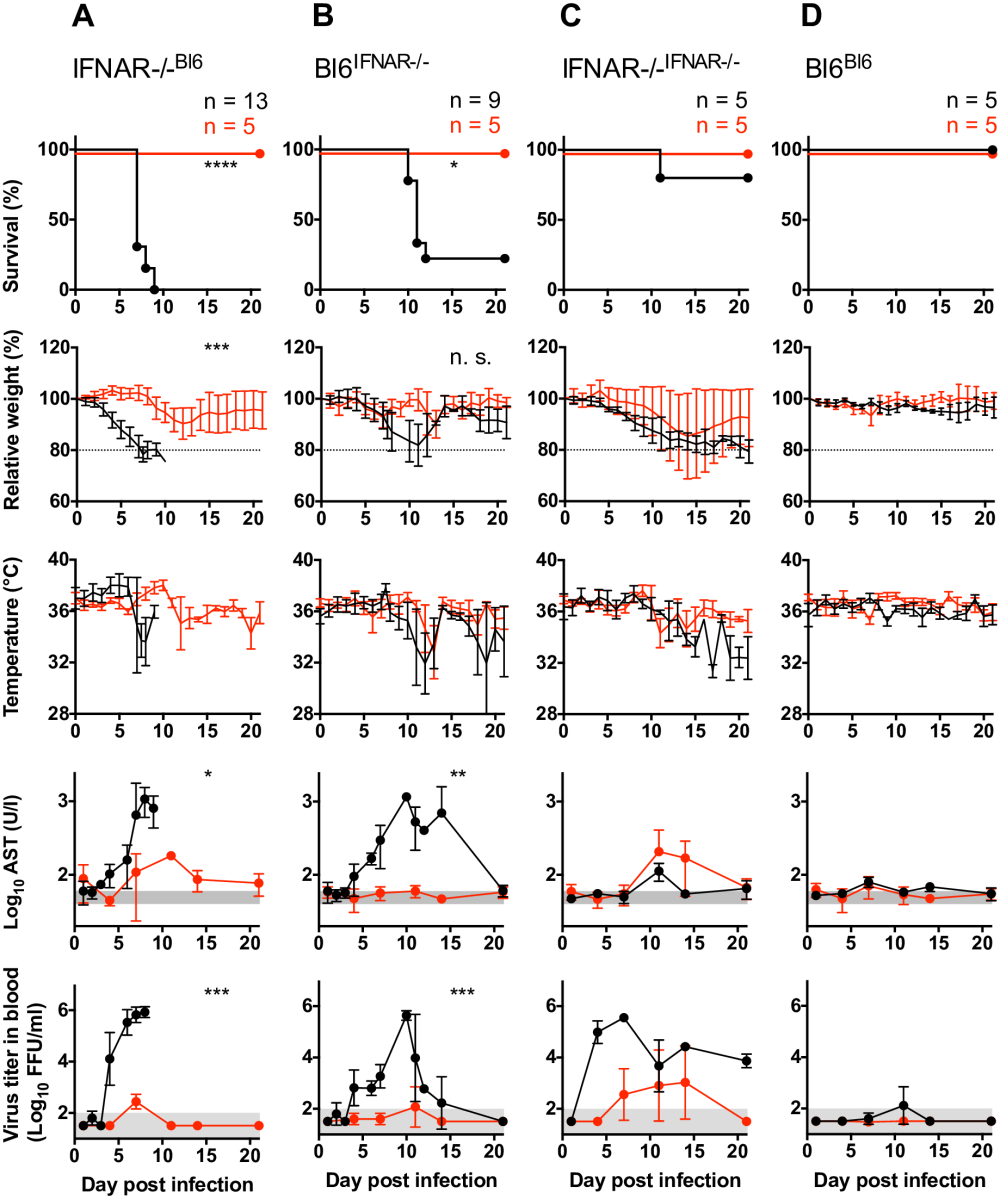
Chimeric mice are less susceptible to non-pathogenic arenaviruses. Chimeric IFNAR^-/- Bl6^ mice (A), Bl6 ^IFNAR-/-^ mice (B), IFNAR^-/- IFNAR-/-^ mice (C) or Bl6 ^Bl6^ mice (D) were inoculated i.p. with 1,000 FFU LASV Ba366 (in black, see Fig 1) or MORV (in red). Survival, relative weight loss, changes in temperature, AST activity and viremia in blood were measured. The normal range for AST and the limit of detection for the virus titer in blood are shaded in gray. Mean and standard deviation are shown. Statistical analysis was done as indicated in Material and Methods.

Within the *Arenaviridae* family of viruses some members cause severe hemorrhagic fever in humans (e. g. LASV, Machupo, Junin, Lujo virus), while others are seemingly low- or non-pathogenic in humans (e. g. Mopeia, Mobala, Morogoro virus). To test whether our mouse model reflected the virulence associated to human pathogenic arenaviruses, the morbidity and mortality of LASV in the chimeric mice was compared with that of Morogoro virus (MORV), a *Mastomys natalensis*-borne non-pathogenic African arenavirus closely related to LASV [22]. In IFNAR^-/- B6^ mice MORV infection caused only mild weight loss and moderated increase of serum AST, with all mice fully recovering from infection (Fig 3A). Similar results were obtained in the IFNAR^-/- IFNAR-/-^ chimeras with survival of 100% of infected mice (Fig 3C). In both B6 ^B6^ and B6 ^IFNAR-/-^ chimeric mice, no signs of any morbidity after MORV infection were detected (Fig 3B and 3D). These results indicated that MORV was not pathogenic for any of the tested mouse models, and was effectively cleared from infected mice even in the total absence of IFN-I signaling. Furthermore, these results also indicated that the mortality associated to chimeric mice infected with LASV, reflected pathophysiological mechanisms associated to LASF and not other arenavirus-induced disease. This was in agreement with the differences between pathogenic and non-pathogenic arenaviruses in humans.

### Moderated inflammation is observed in both LASV and MORV-infected mice

**Fig 4:**
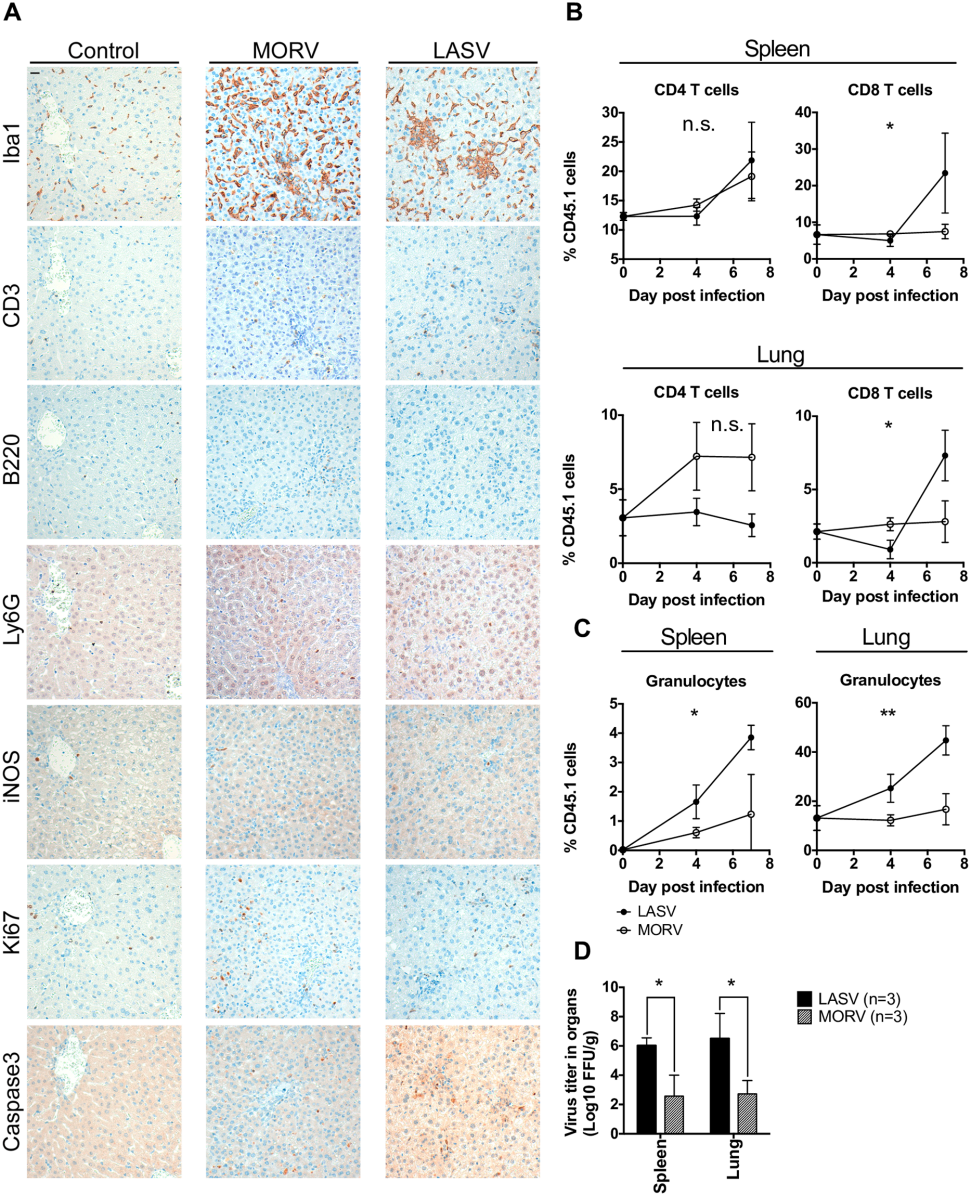
Inflammation in response to LASV vs MORV infection. (A) Chimeric IFNAR^-/- Bl6^ mice were inoculated with 1,000 FFU LASV Ba366, MORV or mock infected. 7 days p. i. liver sections were processed for immunohistochemical staining using the indicated antibodies. Mice were inoculated i. p. with 1,000 FFU LASV or MORV and euthanized at day 0, 4 and 7 p. i. (n = 3). Changes in cellularity of CD4 and CD8 T cells (B) and granulocytes (C) were analyzed using flow cytometry. Mean and standard deviation are shown. Differences in the curves were analyzed via Two-Way ANOVA. (D). Mice were inoculated i. p. with 1,000 FFU LASV Ba366 or MORV and euthanized at day 7 p. i. The virus titers in spleen and liver were determined. Mean and standard devation are shown. Statistical analysis was done using Mann-Whitney non-parametric test.

Since the chimeric IFNAR^-/- B6^ mouse model showed a high degree of susceptibility to LASV but was entirely resistant to MORV, we reasoned that this model was suitable to dissect pathophysiological mechanisms associated to LASF. To test this hypothesis IFNAR^-/- B6^ mice were infected with LASV or MORV and evaluated for tissue-specific pathological findings. Infected mice as well as mock-infected mice were euthanized at day 7 post-infection which coincided with the peak of LASV viremia (Fig 1B). Due to the important involvement of kidney and liver failure in human LASF [3], infected kidney and liver sections were compared with those from mock-infected mice. Immunostaining using anti-Iba1 antibody, a marker of activated monocytes and macrophages [8], showed infiltration of these inflammatory leukocytes in mice infected with either virus (Fig 4A and S3 Fig). Further analysis of liver sections revealed additional similarities between the pathology features caused by both viruses at the peak of viremia, with upregulation of T cell lymphocyte numbers, infiltration of granulocytes, moderated upregulation of inducible nitric oxide synthase (iNOS) and low overall proliferation (Fig 4A). Thus, it seemed that both viruses caused similar degree of inflammation in liver and kidneys, and suggested that more subtle differences in the host immune response were responsible for the radical differences observed in virus-associated morbidity and mortality.

Previous studies have shown that alterations of the lymphoid and non-lymphoid tissue cellularity due to infiltrating antigen-presenting cells and CD8 T cells, are related with the efficacy of the host antiviral response to a variety of viruses [23,24]. Thus, we next evaluated the cellularity of different leukocyte populations over the course of LASV and MORV infection in both lymphoid tissues (spleen) and peripheral tissues (lung). At late time points after infection (day 7), LASV-infected mice showed a significant increase in the frequency of CD8 T but not CD4 T cells compared to MORV-infected mice in both spleen and lungs (Fig 4B). In addition, a significant increase in the frequency of granulocytes, and inflammatory Ly6C^hi^ monocytes was observed (Fig 4C and S4 Fig). These changes in cell frequencies were related with significantly higher virus titers in LASV-infected mice compared with their MORV-infected counterparts (Fig 4D). These results pointed out at CD8 T cells and inflammatory myeloid cells as putative mediators of LASV immunopathology and supported the idea that infiltrating inflammatory cells may support LASV replication.

### Depletion of myeloid cells does not alleviate LASV immunopathology

**Fig 5:**
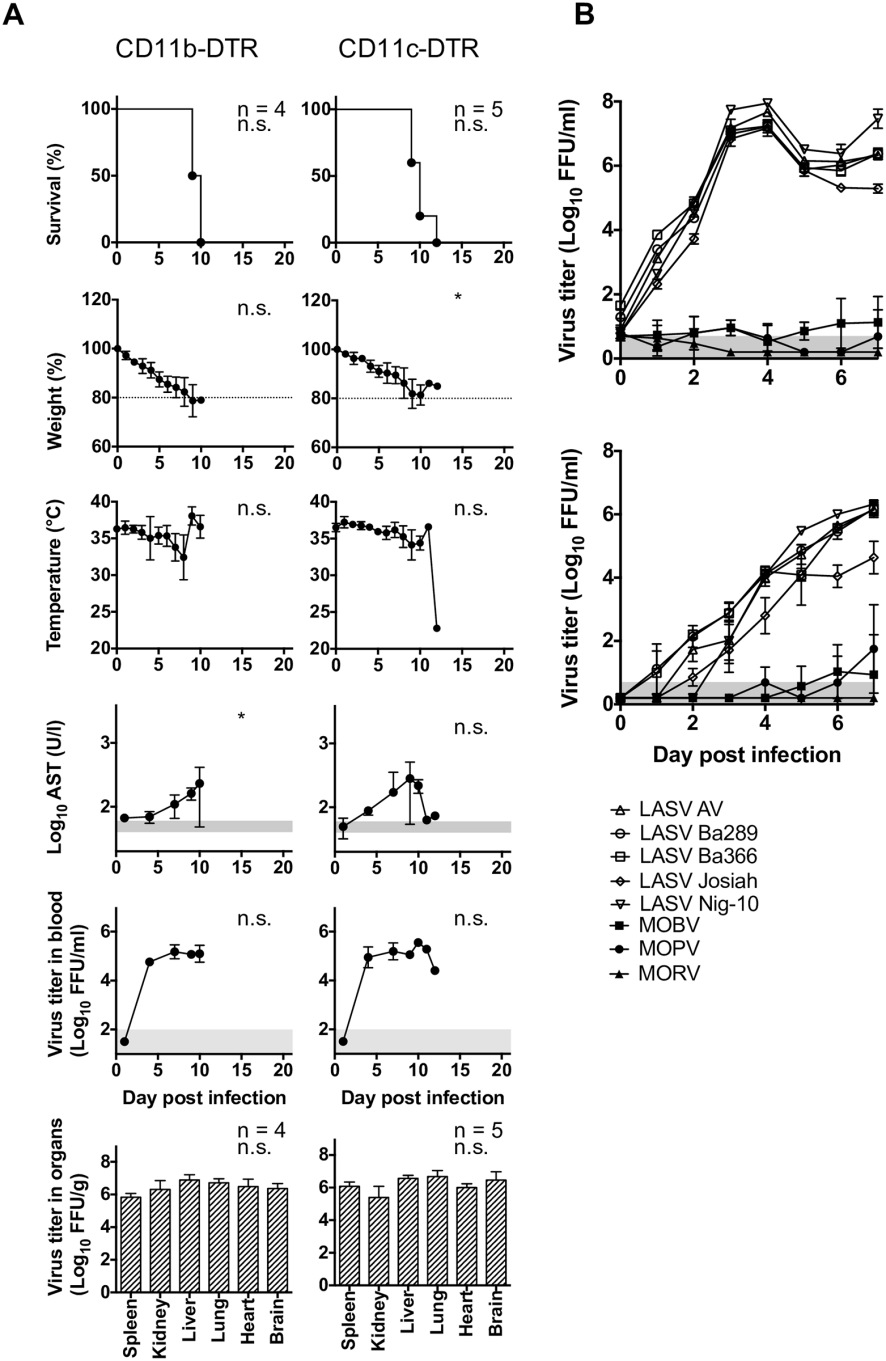
Depletion of myeloid cells has no impact on the disease. (A) Mice were depleted for CD11b or CD11c cells by treating IFNAR^-/- CD11b^ or IFNAR^-/- CD11c^ mice with diphtheria toxin prior to infection. The mice were inoculated i. p. with 1,000 FFU LASV Ba366. Mice were monitored for survival, weight loss and temperature. AST activity, virus titer in blood and virus titer in organs were measured at the indicated time points. The normal range for AST and the limit of detection for the virus titer in blood are shaded in gray. Mean and standard deviation are shown. Statistical analysis was done as indicated in Material and Methods. (B) Bone marrow derived macrophages (upper graph) and dendritic cells (lower graph) were inoculated with different pathogenic and non-pathogenic arenaviruses with a MOI of 0.01. Aliquots of the cell culture supernatant were taken daily and analyzed for infectious virus particles (n = 3).

A significant accumulation of Ly6C^hi^ inflammatory monocytes was observed over the course of LASV infection (S4 Fig). This is a myeloid population with an important immunopathologic role in other viral infections such as pulmonary influenza [24,25]. Thus, we reasoned that specific depletion of monocytes could serve to alleviate LASV immunopathology. To test this, chimeric mice were generated in which irradiated IFNAR^-/-^ recipients were transplanted with bone marrow progenitor cells from CD11b-diphtheria toxin (DT) receptor (DTR) transgenic donor mice, or CD11c-DTR donor mice. In these mice, administration of DT kills CD11b-expressing monocytes and macrophages, or CD11c-expressing dendritic cells respectively, thus allowing evaluation of the specific functions of these myeloid populations [26,27]. In chimeric IFNAR^-/- CD11b-DTR^ and IFNAR^-/- CD11c-DTR^ mice more than 95% of monocytes and dendritic cells were from donor origin and were depleted by intraperitoneal administration of DT (S5 Fig). Perhaps surprisingly, depletion of CD11b- and CD11c-expressing cells did not prevent death from LASV infection, nor did it prevent viremia and virus replication in peripheral organs (Fig 5A). These findings indicated that despite the infiltration of inflammatory myeloid cells observed in the organs of LASV-infected mice, these cells were not responsible for virus-associated immunopathology. However a reduction in the levels of cell damage (as indicated by reduced levels of serum AST) was observed and also viremia in CD11b-depleted mice compared with IFNAR^-/- B6^ chimeras (Figs 5A and 1). These results suggested that, at least to some extent, CD11b^+^ cells could play a role supporting viral replication and dissemination. To test this hypothesis, monocyte-derived dendritic cells (moDCs) were differentiated *in vitro* via incubation of bone marrow progenitor cells obtained from C57BL/6 mice with granulocyte-macrophage colony-stimulating factor (GM-CSF). *In vitro*-differentiated mo-DCs were productively infected with LASV strains belonging to various lineages, but were entirely refractory to MORV infection as well as infection with other non-pathogenic arenaviruses such as Mobala virus (MOBV) and Mopeia virus (MOPV) (Fig 5B). Together, our findings indicated that productive infection of inflammatory myeloid cells may play a role on systemic LASV dissemination or virus replication in peripheral tissues (see Fig 4D), but that the infiltration of these cells in peripheral tissues does not influence LASV immunopathology.

### Depletion of CD8 T cells rescues mice from death by LASV infection

**Fig 6:**
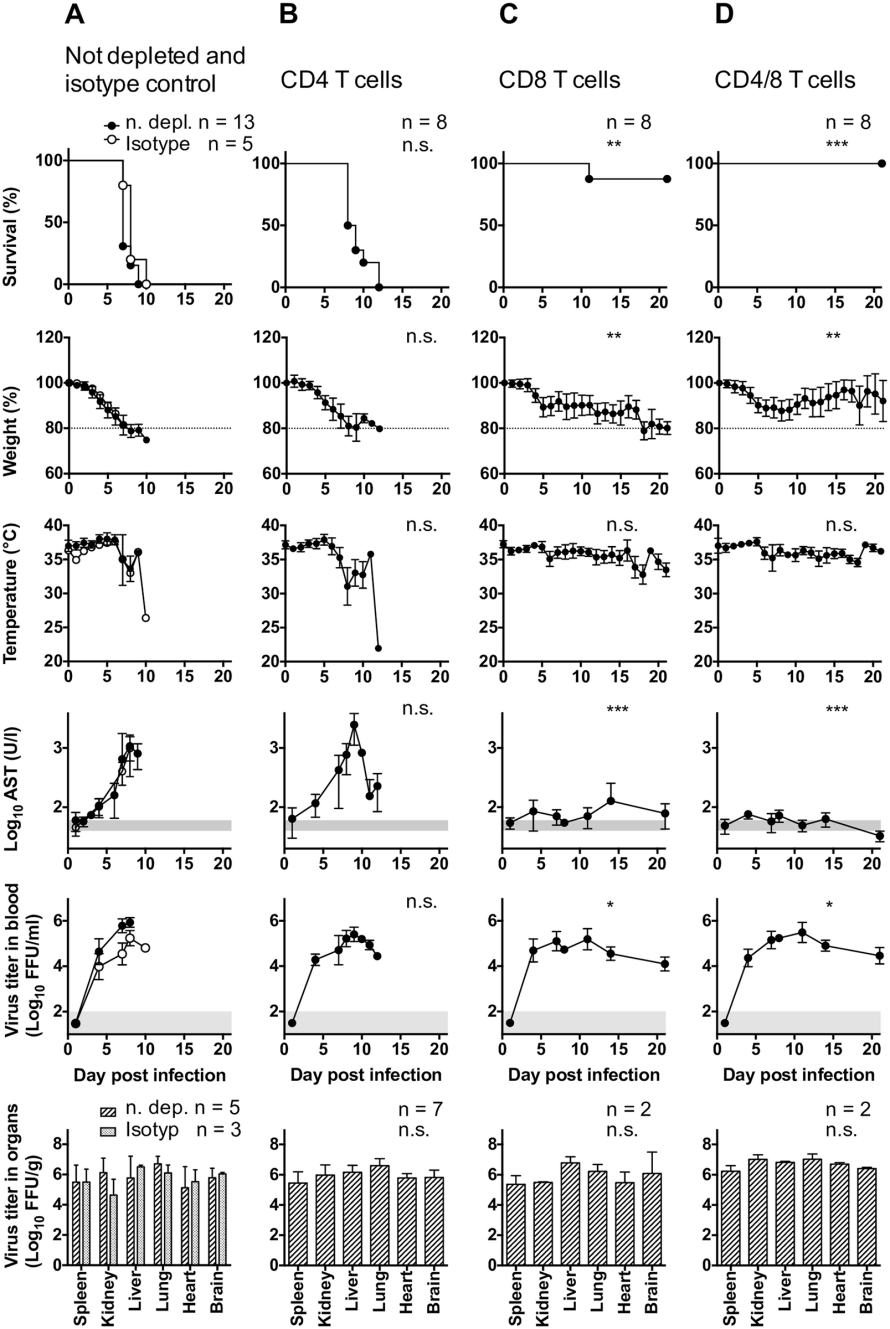
Depletion of rescues LASV-infected mice. IFNAR^-/- Bl6^ mice were depleted for CD4 T cells (B), CD8 T cells (C) or both (D) with anti-CD4 or anti-CD8 antibodies. Control mice (A) remained untreated (Not depleted) or received an isotype control antibody (isotype control). All groups were inoculated i. p. with 1,000 FFU of LASV Ba366. Mice were monitored for survival, weight loss and temperature. AST activity, virus titer in blood and virus titer in organs were measured at the indicated timepoints. The normal range for AST and the limit of detection for the virus titer in blood are shaded in gray. Mean and standard deviation are shown. The T cell depleted groups were compared to the undepleted group. Statistical analysis was done as indicated in Material and Methods.

Next the role of T cells on LASV pathogenesis was examined. IFNAR^-/- B6^ chimeric mice were depleted of CD4 and CD8 T cells via i.p. injection of monoclonal antibodies (clones YTS191 and YTS169 respectively) starting three days before infection with LASV (S5B Fig). As controls, mice were injected with isotype antibodies or left untreated. As shown in Fig 6A, isotype and non-treated mice displayed features of severe LASF with high viremia, elevated AST, virus replication in peripheral organs and death within ten days post-infection. This was also the case for mice depleted of CD4 T cells, which indicated that these cells alone were not responsible for immunopathology (Fig 6B). Strikingly, depletion of CD8 T cells rescued 87,5% of mice from LASV-induced death, despite the fact that elimination of these cells resulted in persistent viremia (Fig 6C). Mouse survival was also associated with a marked reduction of cell damage, as indicated by low levels of serum AST. Finally, simultaneous depletion of CD4 and CD8 T cells resulted in 100% survival of chimeric mice infected with LASV (Fig 6D), which demonstrated a chief role of CD8 T cells on LASV-induced immunopathology, and also a putative contribution of CD4 T cells, albeit at lower levels. Of note, neither depletion of CD4 T cells nor CD8 T cells resulted in changes on the levels of virus replication in peripheral organs compared to non-depleted controls (Fig 6B–6D). These results indicated a key role of T cell-mediated immunopathology on LASF severity in our model, and a poor capacity of T cells to contribute to viral clearance.

### CD8 T cells drive vascular leakage during LASV infection

**Fig 7:**
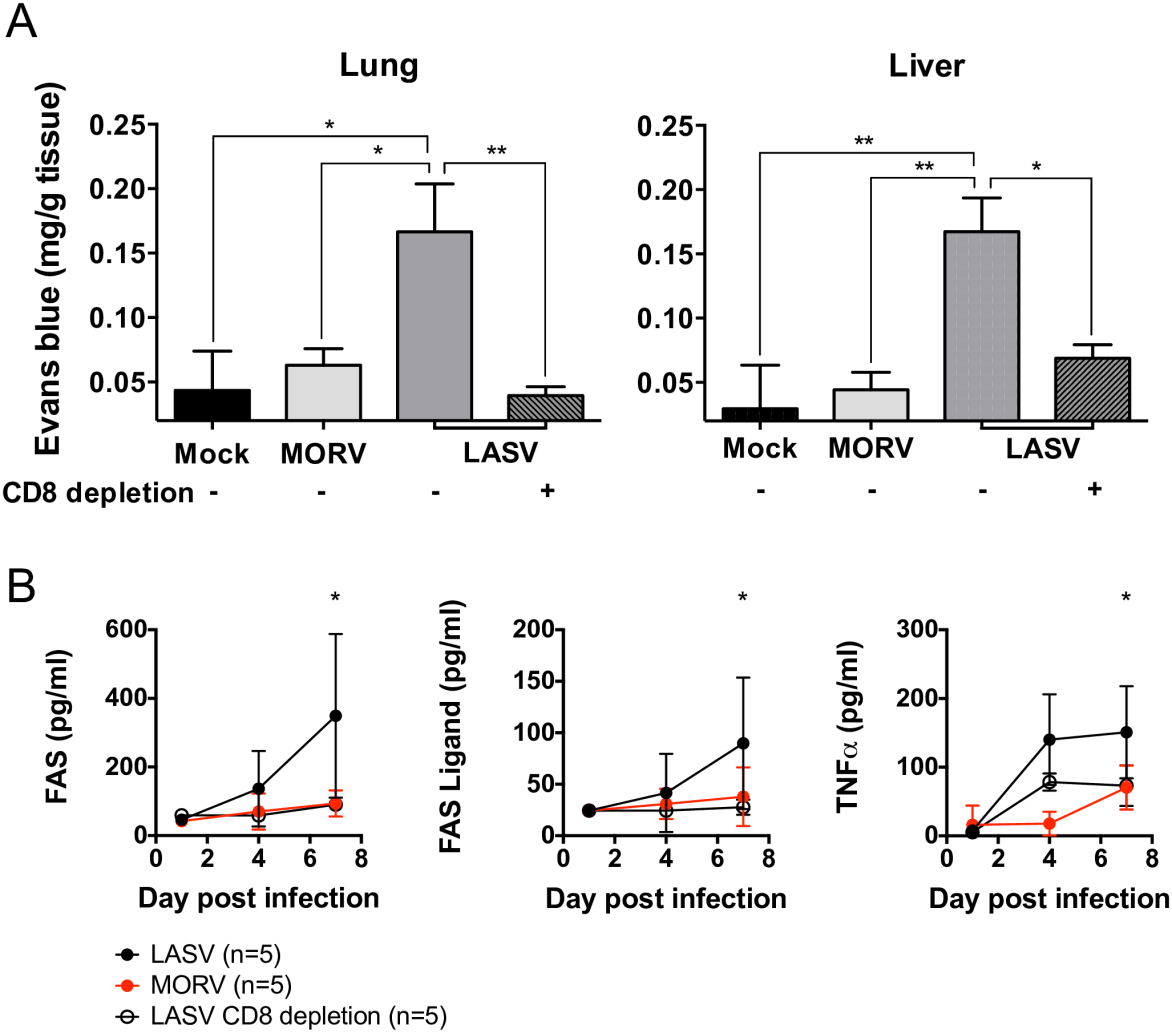
CD8 T cells drive vascular leakage during LASV infection. (A) IFNAR^-/- Bl6^ mice were depleted of CD8 T cells with anti-CD8 antibody or remained untreated as indicated in the figure. Each group consisted of n = 5 mice. Mice were inoculated i. p. with 1,000 FFU MORV, LASV Ba366 or were Mock infected with PBS. Mice were injected intravenously with 100 μl Evans Blue on day 7 p. i., euthanized and perfused with NaCl. Lung and Liver were analyzed for vasculear leakage. Mean and standard deviation are shown. Differences in vasculear leakage were analyzed using Mann-Whitney non-parametric test. (B) Plasma concentrations of FAS, FAS ligand and TNFα were determined via Luminex in samples obtained at days 1, 4 and 7 p. i. from CD8 depleted or undepleted IFNAR^-/- Bl6^ mice, infected with MORV (in red) or LASV Ba366 (in black). Mean and standard deviation are shown. Differences in the curves were analyzed using Kruskal-Wallis test and Dunn’s post test.

A common feature of viral hemorrhagic fevers including LASF is vascular leakage which in the case of LASF has been associated to edema and hematological disfunction [3,15,28]. CD8 T cells have been strongly correlated with vascular leakage in other acute infection models such as cerebral malaria [29,30] and LCMV, in the latter, due to direct killing of infected endothelial cells [31]. Thus, we reasoned that CD8 T cell-mediated vascular leakage could explain mechanistically their association with disease severity in our model. To test this hypothesis we administered Evans Blue (EB), a dye with high affinity to serum albumin, to IFNAR^-/- B6^ mice at the peak of infection with LASV or MORV. Photometrical quantification of EB in the lung and liver of infected mice indicated significantly higher vascular leakage in LASV-infected mice compared with MORV-infected mice (Fig 7A). Strikingly, vascular leakage in peripheral organs of LASV-infected mice was entirely prevented via depletion of CD8 T cells (Fig 7A). In agreement with these findings being correlated with CD8 T cell-mediated cytotoxicity, the serum levels of FAS and FAS-L were significantly higher in LASV-infected mice compared to MORV-infected mice, and were significantly reduced after CD8 T cell depletion (Fig 7B). These results demonstrated that LASV lethality was associated to CD8 T cell mediated immunopathology which was mechanistically correlated with increased vascular permeability.

## Discussion

Previous studies have described several animal models of LASF including immunodeficient mice, guinea pigs and non-human primates (NHPs) [11,32]. Of these models, only NHPs displayed immunocompetence as well as human features of LASF (i. e. liver tropism) [11,13]. However, due to the limited availability, lack of reagents, and high costs associated to NHP research, this model is not optimal for the dissection of the physiological mechanisms involved in LASF immunity and pathogenesis.

Here we present a chimeric mouse model generated by bone marrow transplantation, in which restriction of IFN-I deficiency to either the radio-resistant or the hematopoietic compartment resulted in high susceptibility to LASV. These results are in agreement with the role of IFN-I-induced antiviral state in restricting early LASV infection [18,33], and suggest that viral replication in discrete IFN-I-deficient cell compartments is sufficient to establish disease. Moreover, our study also suggested that not only dendritic cells and monocyte-derived macrophages of hematopoietic origin, but also other radio-resistant or stromal cell types may be susceptible to LASV infection *in vivo*. It is worth noting that in our chimeric mouse model, putative LASV target cells such as tissue-resident macrophages, alveolar macrophages, Kupffer cells and Langerhans cells, [8,9] are host-derived due to the fact that these cells are originated from yolk sac, and not hematopoietic progenitors [34]. The importance of early replication of LASV in susceptible cells for the establishment of infection, is also supported by the finding that LASV but not non-pathogenic arenaviruses such as MORV, MOBV and MOPV, was able to replicate in mouse monocyte-derived dendritic cells *in vitro*.

Interestingly, despite the fact that LASV but not MORV was lethal for chimeric mice, histopathological studies revealed moderated liver damage–a prominent feature of LASV infection in NHPs and in humans [35]–in either infection. A possible explanation for this observation is that lack of IFN-I signaling in hematopoietic or radio-resistant cells may reduce IFN-I-mediated oxidative damage of hepatocytes [36]. This is also consistent with the finding that STAT-1 mice infected with LASV also showed lower levels of circulating serum aminotransferases compared to NHPs and humans [18]. As previously reported in other animal models as well as in humans, LASV infection resulted in moderated inflammation in several peripheral organs, with infiltration of inflammatory monocytes/macrophages, T cells and granulocytes. Similar findings were found in chimeric mice infected with MORV. However, specific depletion of antigen-presenting cells such as monocytes and dendritic cells in LASV-infected mice, only caused moderated reduction of viremia, and did not alleviate LASV-induced disease. Conversely, depletion of CD8 T cells was sufficient to alleviate LASV-induced morbidity and mortality in IFNAR^-/- B6^ mice. The importance of T cells as drivers of LASV-mediated immunopathology in our model does not reconcile easily with the finding that T cell activation is necessary for early control of LASV replication in NHPs [13]. However, our previous studies utilizing mice expressing human HLA also demonstrated that T cells were required for early viral clearance but if they failed to do so–due for example to high viral loads–they worsened the ensuing disease process [8,13]. Similar paradoxical findings indicating both detrimental and protective effects of T cells have been found in many models of viral infection including influenza virus [37], dengue virus [38], LCMV [31] and hantavirus [39]. These findings suggest that defects in T cell homeostasis mechanisms modulating the balance between protective and immunopathogenic T cell responses may play an important role in many viral infections. Thus, perhaps, the differences found in our model and NHPs only reflect partial observations of a complex physiological process. Further experiments utilizing different doses and routes of infection in our model as well as T cell depletion assays in NHPs may help to further evaluate similarities between our model and the NHP model. Of note, simultaneous depletion of CD8 and CD4 T cells further alleviated LASV-induced disease, a finding that may reflect CD4-mediated cytotoxicity as described for other acute viral infections [40], or perhaps the important role of Foxp3^+^ regulatory CD4 T cells (T_regs_) at preventing arenavirus-mediated immunopathology [41].

In our model, lethality was not restricted to IFNAR^-/- B6^ chimeras, and B6 ^IFNAR-/-^ mice were also highly susceptible to LASV infection with 75% lethality. We showed that IFN-I competence in either compartment was correlated with increased levels of serum pro-inflammatory mediators in agreement with IFN-I as a key inducer of antiviral pro-inflammatory signals [15,42,43]. A number of previous reports have identified innate or bystander CD8 T cell activation via cytokine-mediated, TCR-independent stimulation, which results in rapid production of IFN-γ and TNF-α as well as cytotoxicity [44–46]. Indeed, we observed significantly higher levels of serum IFN-γ and TNF-α at the peak of LASV infection in IFNAR^-/- B6^ and B6 ^IFNAR-/-^ mice compared to control chimeras. Although preliminary, these results strongly suggest that cytokine-mediated CD8 T cell activation may be an important mechanism of LASF pathogenesis and warrants further investigations. Additionally, the levels of FAS, FAS-L and granzyme B were significantly upregulated in IFNAR^-/- B6^ mice which suggests that T cell-mediated cytotoxicity may account, at least to some extent, for the increased lethality in this model.

Previously, mice lacking functional STAT-1 were shown to reproduce features of human LASF also present in our model such as systemic virus dissemination, elevated serum aminotransferases and hypothermia during the late stages of disease [18]. Despite lacking functional IFN-I signaling, these mice were highly susceptible to LASV and developed severe disease and post-recovery sequelae such as hearing loss which is commonly found in LASF survivors [47]. Interestingly, the severity of infection was suggested to be dependent in this model at least partially to loss of STAT-1-dependent regulation of T cell homeostasis [48,49] which resulted in T cell infiltration and damage to the cochlear nerve [47]. These findings further strengthen the notion that T cell-mediated immunopathology is an important pathophysiological feature of LASF.

As CD8 T cell-mediated cytotoxicity has been directly linked to vascular leakage [31], we investigated the correlation between CD8 T cell immunity and edema, a prominent feature of human LASF and other arenaviral hemorrhagic fevers [3,50,51]. We demonstrated that LASV but not MORV-infected mice suffered vascular leakage leading to edema in lungs and liver, and that this phenotype was entirely alleviated by CD8 T cell depletion. These results strongly suggest association between CD8 T cell immunopathology, vascular leakage and death, and reflect important differences in our model between infection with non-pathogenic and pathogenic arenaviruses.

Our system provides a platform for testing arenavirus pathogenicity and strongly suggests that T cell-mediated immunopathology plays an important role in LASF pathophysiology. However, whether or not our findings reflect human disease remains to be determined. Unfortunately, to date there is little insight into the pathophysiology mechanism of human LASF which requires adequate clinical studies in the field. Elucidation of the role of T cells in disease severity in humans could provide a rationale for immunotherapeutic interventions against LASF.

## Supporting Information

S1 Fig Chimeric mice and transplantation controls. (A) Donor cell engraftment in peripheral blood of IFNAR^-/- Bl6^ and Bl6^IFNAR-/-^ chimeric mice was evaluated by flow cytometry as indicated. (B) Non-infected IFNAR^-/- Bl6^ and Bl6 ^IFNAR-/-^ mice were monitored for survival and morbidity signs such as relative weight loss, body scoring, changes in temperature and cellular damage (serum levels of AST). The normal range for AST is shaded in gray. Mean and standard deviation are shown.
(PDF)

S2 Fig Susceptibility of chimeric mice to different LASV strains. Chimeric IFNAR^-/- B6^ mice were inoculated i. p. with 1,000 FFU of the indicated LASV strains and morbidity and mortality was assessed over the course of infection. The normal range for AST and ALT and the limit of detection for virus titers in blood are shaded in gray. Mean and standard deviation are shown. All strains were uniformly lethal within the first 11 days post-inoculation.
(PDF)

S3 Fig Comparative pathology of MORV and LASV in kidneys of infected mice. Chimeric IFNAR^-/- Bl6^ mice were inoculated with 1,000 FFU LASV Ba366, MORV or mock infected. 7 days p. i. kidney sections were processed for immunohistochemical staining using the indicated antibodies.
(PDF)

S4 Fig Inflammation associated to LASV infection in chimeric mice. (A) Flow cytometric analysis of the cellularity of the hematopoietic compartment in spleen and lung of IFNAR^-/- B6^ mice over the course of infection. Ly6C^hi/low^ cells correspond to CD45^+^ CD11b^+^ CD11c^+^ CD209^+^ SSC^low^ Ly6G^-^ Ly6C^hi/low^ cells consistent with inflammatory monocytes. (B) Cellularity of lungs of LASV and MORV-infected IFNAR^-/- B6^ mice at days 0, 4 and 7 post-infection. C. Representative plots showing infiltration of Ly6C^hi^ inflammatory monocytes in the lung of mice infected with either LASV or MORV at the indicated time points post infection.
(PDF)

S5 Fig Depletion of myeloid cells and T cells in mice. (A) DT-based depletion of cells expressing CD11b in peripheral blood of mice. Depletion was accomplished by i.p injection of 0.2 μg of diphtheria toxin (DT). Plots show depletion levels at day on day 1 post-DT administration. (B) Depletion of CD8 and CD4 T cells was achieved by i.p. administration of monoclonal antibodies. Plots indicate levels of depletion of T cells at day 1 post-administration.
(PDF)
