# Supplementary figures and images for: Chimeric Mice with Competent Hematopoietic Immunity Reproduce Key Features of Severe Lassa Fever
Source: PLoS Pathog. 2016 May 18;12(5):e1005656. doi: 10.1371/journal.ppat.1005656 (PMC4871546; doi:10.1371/journal.ppat.1005656)

**A**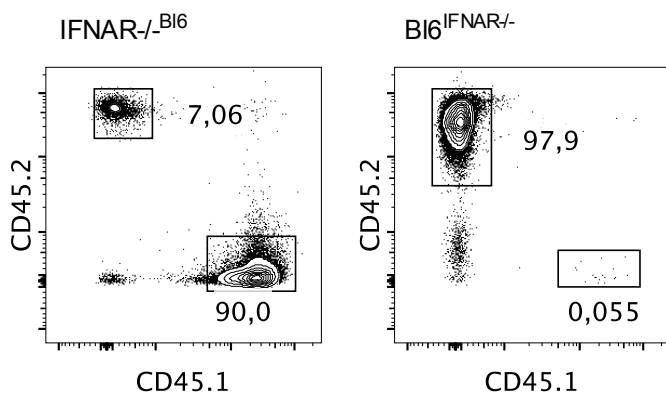**B**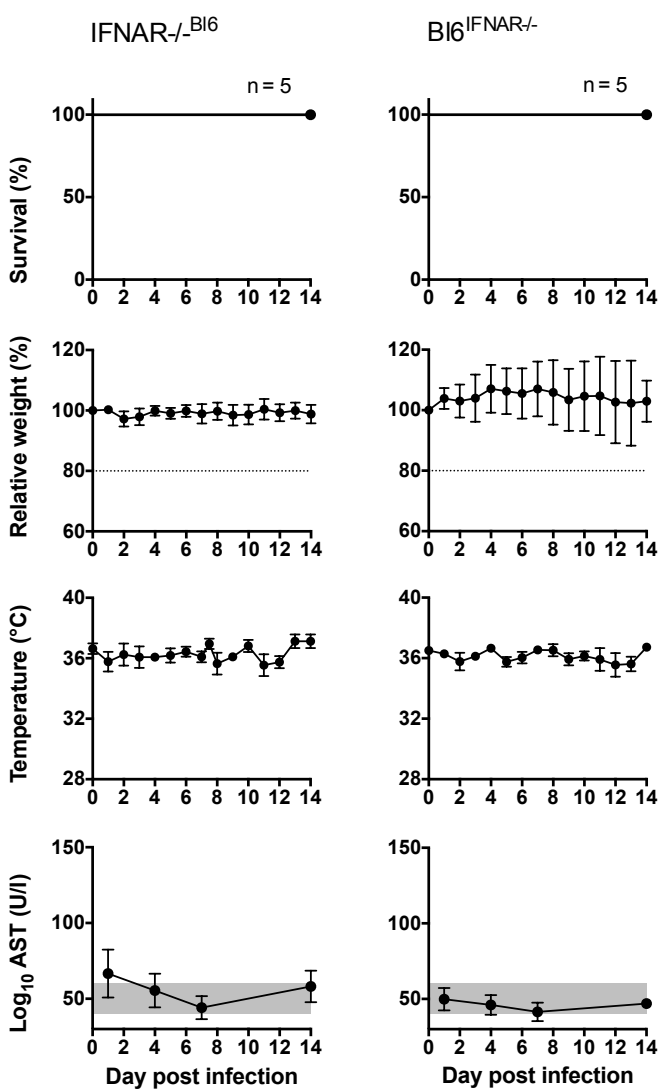

Supplement: S1 Fig — (A) Donor cell engraftment in peripheral blood of IFNAR-/- Bl6 and Bl6IFNAR-/- chimeric mice was evaluated by flow cytometry as indicated. (B) Non-infected IFNAR-/- Bl6 and Bl6 IFNAR-/- mice were monitored for survival and morbidity signs such as relative weight loss, body scoring, changes in temperature and cellular damage (serum levels of AST). The normal range for AST is shaded in gray. Mean and standard deviation are shown. (PDF) [file ppat.1005656.s001.pdf]

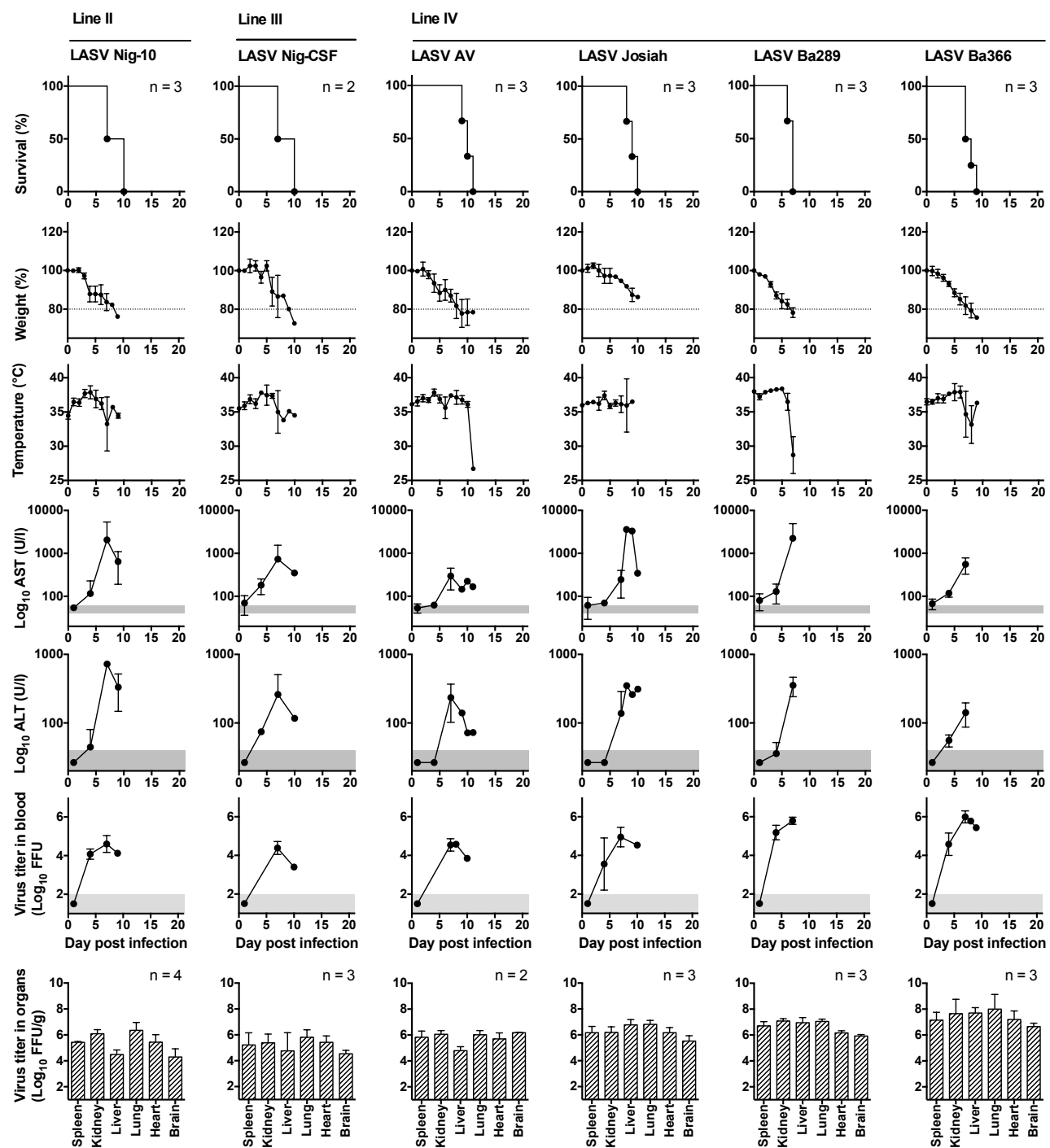

Supplement: S2 Fig — Chimeric IFNAR-/- B6 mice were inoculated i. p. with 1,000 FFU of the indicated LASV strains and morbidity and mortality was assessed over the course of infection. The normal range for AST and ALT and the limit of detection for virus titers in blood are shaded in gray. Mean and standard deviation are shown. All strains were uniformly lethal within the first 11 days post-inoculation. (PDF) [file ppat.1005656.s002.pdf]

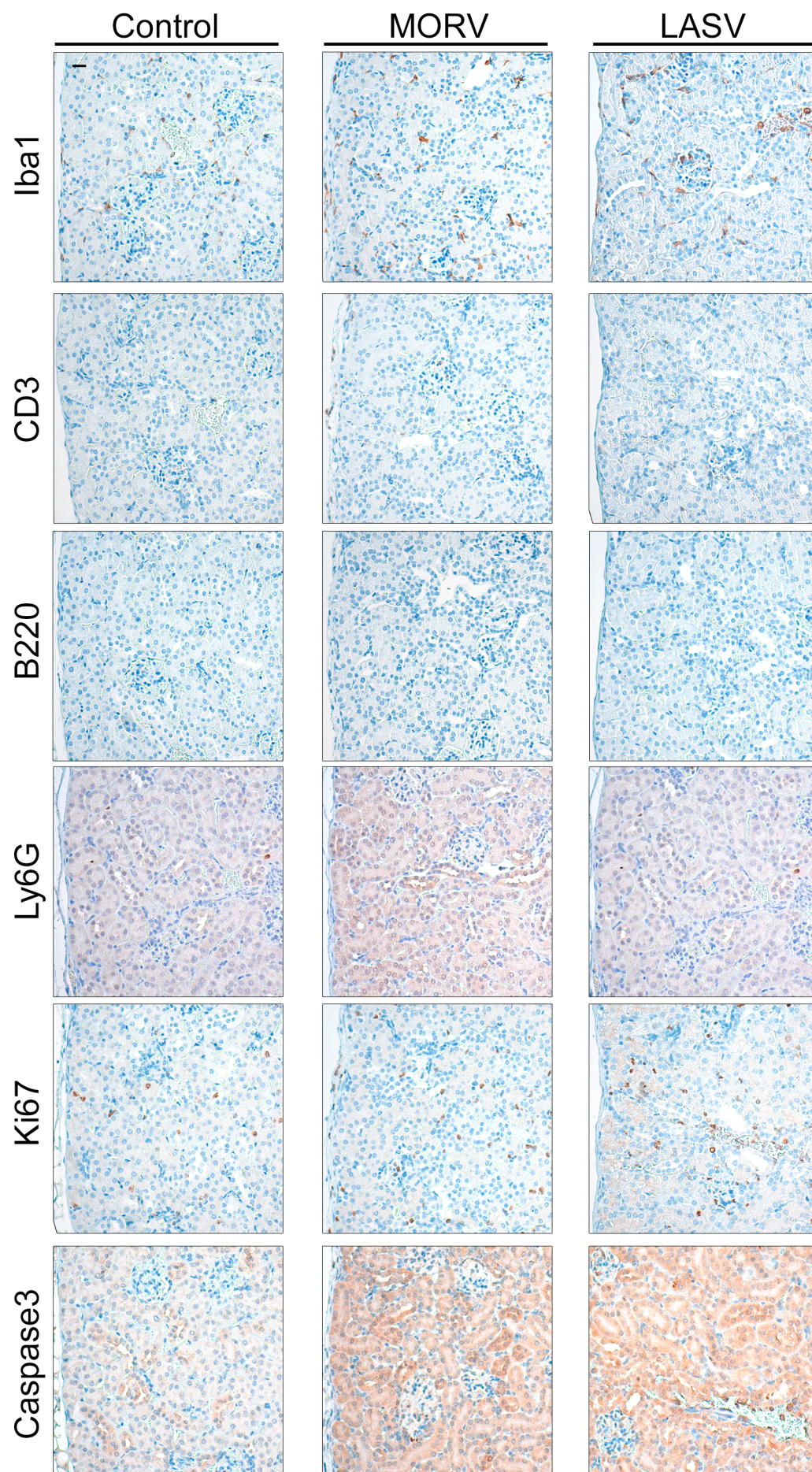

Supplement: S3 Fig — Chimeric IFNAR-/- Bl6 mice were inoculated with 1,000 FFU LASV Ba366, MORV or mock infected. 7 days p. i. kidney sections were processed for immunohistochemical staining using the indicated antibodies. (PDF) [file ppat.1005656.s003.pdf]

A

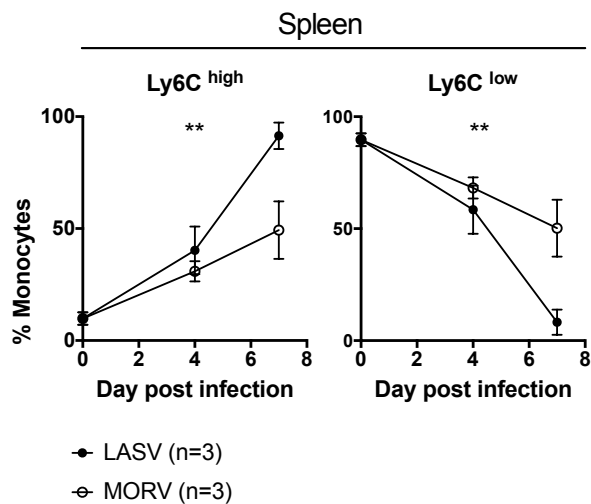

B

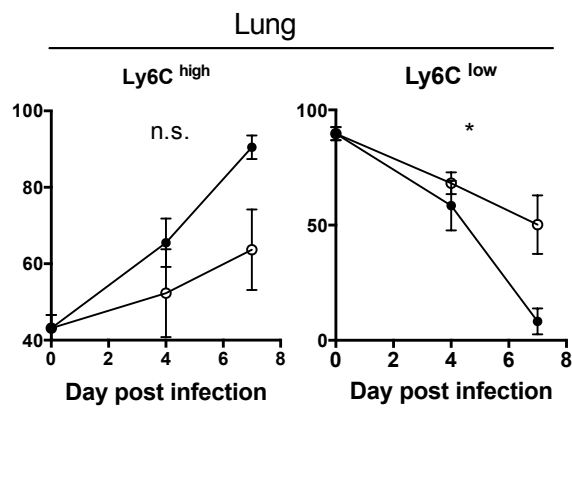

C

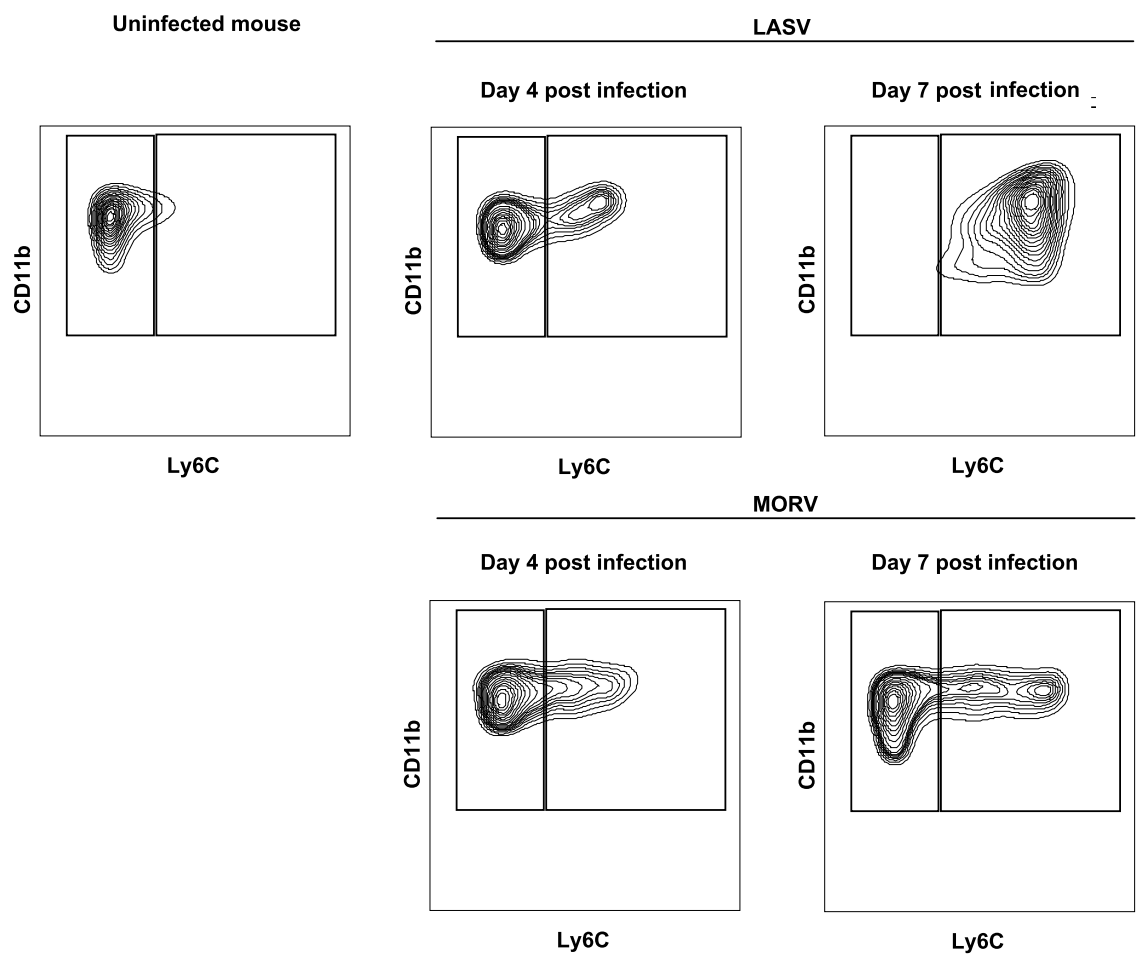

Supplement: S4 Fig — (A) Flow cytometric analysis of the cellularity of the hematopoietic compartment in spleen and lung of IFNAR-/- B6 mice over the course of infection. Ly6Chi/low cells correspond to CD45+ CD11b+ CD11c+ CD209+ SSClow Ly6G- Ly6Chi/low cells consistent with inflammatory monocytes. (B) Cellularity of lungs of LASV and MORV-infected IFNAR-/- B6 mice at days 0, 4 and 7 post-infection. C. Representative plots showing infiltration of Ly6Chi inflammatory monocytes in the lung of mice infected with either LASV or MORV at the indicated time points post infection. (PDF) [file ppat.1005656.s004.pdf]

A Gate: SSC<sup>low</sup> Singlet cell population

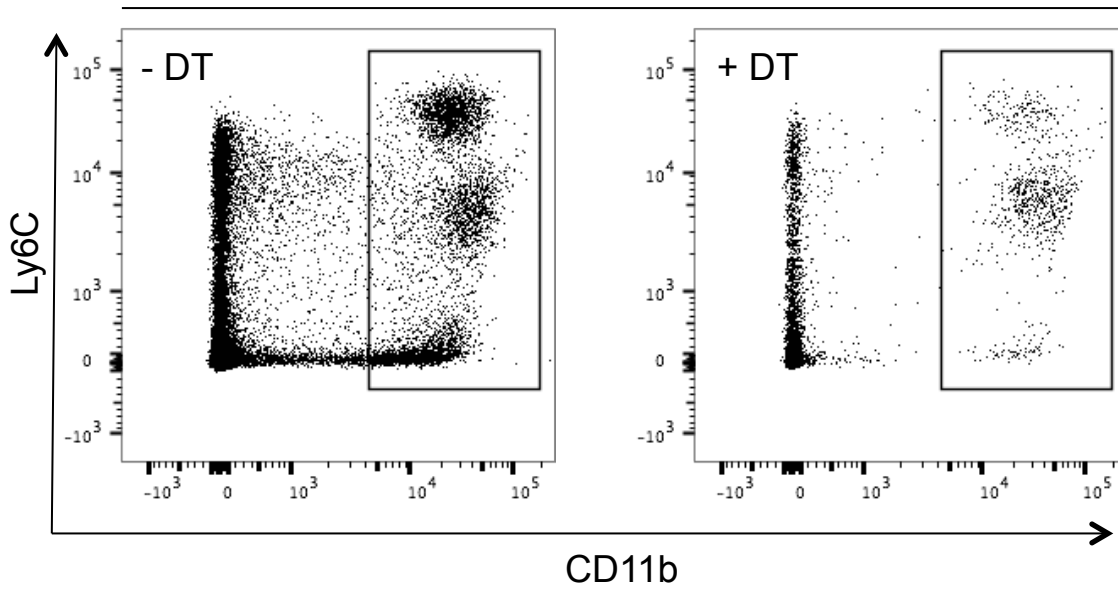

B Gate: CD3<sup>high</sup> Singlet cell population

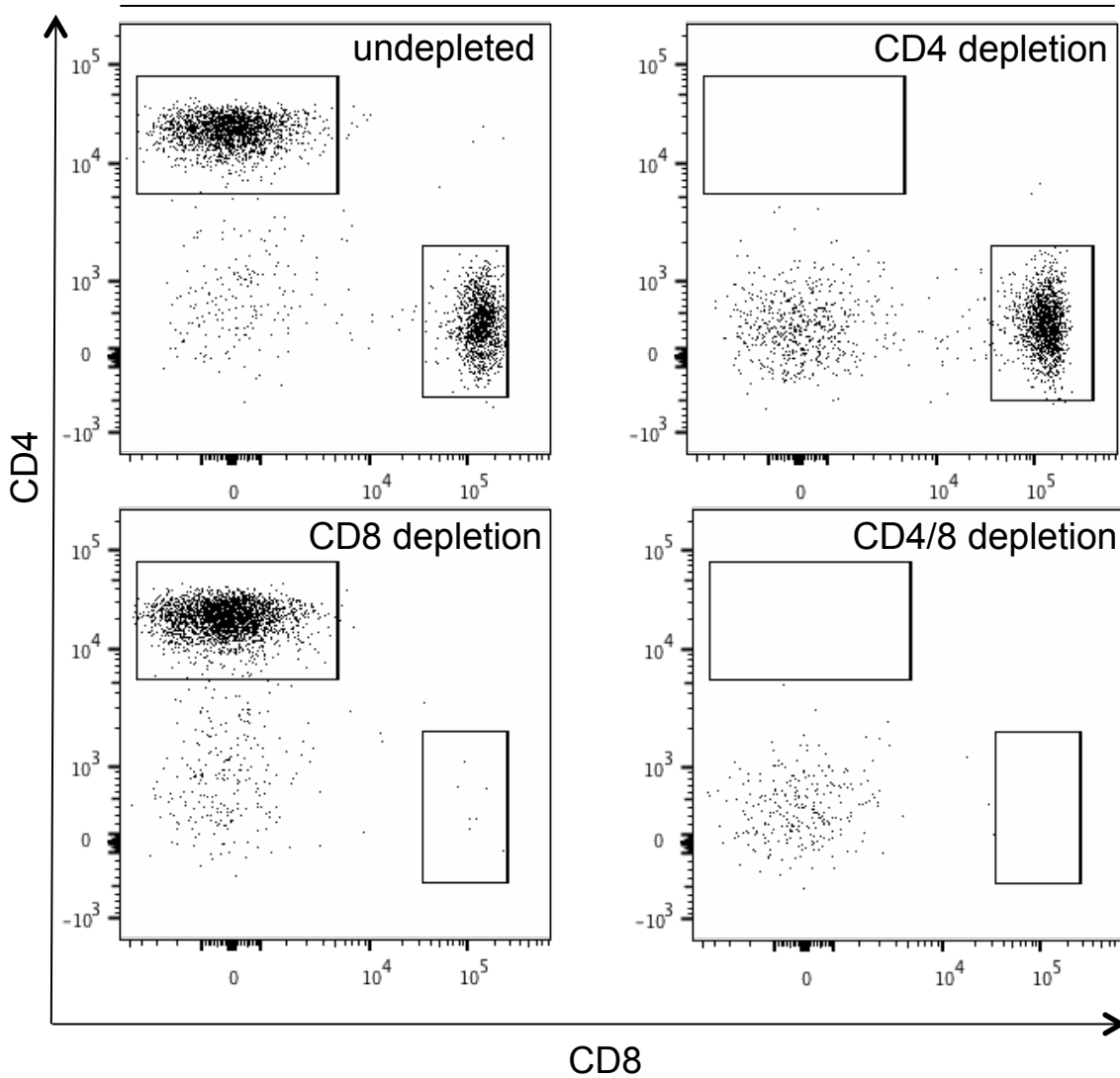

Supplement: S5 Fig — (A) DT-based depletion of cells expressing CD11b in peripheral blood of mice. Depletion was accomplished by i.p injection of 0.2 μg of diphtheria toxin (DT). Plots show depletion levels at day on day 1 post-DT administration. (B) Depletion of CD8 and CD4 T cells was achieved by i.p. administration of monoclonal antibodies. Plots indicate levels of depletion of T cells at day 1 post-administration. (PDF) [file ppat.1005656.s005.pdf]
